# Advanced bilateral glaucoma decades after first-generation Fyodorov ‘collar-button’ anterior–posterior phakic intraocular lens implantation: a case report and clinical management considerations

**DOI:** 10.3389/fopht.2026.1677323

**Published:** 2026-05-04

**Authors:** Monika Sarnat-Kucharczyk, Anna Hitnarowicz, Aleksandra Ziemba, Dorota Pojda-Wilczek, Ewa Mrukwa-Kominek, Sudi Patel

**Affiliations:** 1 Department of Ophthalmology, Faculty of Medical Sciences in Katowice, Medical University of Silesia, Katowice, Poland; 2 Department of Ophthalmology, Professor Kornel Gibiński University Clinical Centre, Katowice, Poland; 3 Student Scientific Association at the Department of Ophthalmology, Faculty of Medical Sciences in Katowice, Medical University of Silesia, Katowice, Poland; 4 Department of Cataract and Refractive Surgery, University Eye Clinic Svjetlost, Zagreb, Croatia

**Keywords:** phakic intraocular lens, Fyodorov collar-button IOL, glaucoma, glaucomatous optic neuropathy, cataract, micropulse transscleral cyclophotocoagulation

## Abstract

**Background:**

Phakic intraocular lenses (pIOLs), used to correct high refractive errors, have since been associated with long-term complications including glaucoma and cataract formation. Elevated intraocular pressure (IOP) in these patients can be challenging to manage, often requiring surgical intervention. Micropulse trans-scleral cyclophotocoagulation (MP-TSCPC) offers a less invasive option for IOP control in refractory cases. This report describes a patient with advanced glaucomatous optic neuropathy decades after undergoing pIOL implantation and the successful use of MP-TSCPC.

**Case presentation:**

A 45-year-old male presented in June 2023 with advanced glaucomatous optic neuropathy following bilateral implantation of a first-generation Fyodorov “collar-button” phakic intraocular lens (pIOL) performed 25 years earlier. The patient experienced worsening vision in both eyes. Visual acuity testing, slit-lamp examination, static visual field (VF) assessment, anterior and posterior segment optical coherence tomography (AS-OCT, PS-OCT), pattern and flash visual evoked potentials (PVEP/FVEP) were performed. Corrected distance visual acuity (CDVA) was 0.9 in the RE and 1.0 in the left eye LE on the Snellen chart. Corrected near visual acuity (CNVA) was 0.5 on the Jaeger chart in both eyes. IOP was 26 mmHg in the RE and 24 mmHg in the LE. Slit-lamp examination revealed the presence of pIOLs with anterior capsular cataracts in both eyes. Fundoscopy showed severe glaucomatous optic neuropathy. Gonioscopy indicated a 30-degree angle width with grade 3 trabecular pigmentation. Visual field testing revealed advanced scotomata. PS-OCT confirmed severe ganglion cell and retinal nerve fiber loss. Performed examinations confirmed bilateral glaucomatous optic nerve atrophy. Despite the use of antiglaucoma medications, IOP remained elevated 3 weeks later measuring 23 mmHg in the RE and 21 mmHg in the LE. Due to persistently elevated intraocular pressure (IOP) despite pharmacological treatment, micropulse transscleral cyclophotocoagulation (MP-TSCPC) was performed in the right eye (RE). Following MP-TSCPC in the RE, the IOP decreased to 16 mmHg. The LE was managed pharmacologically, achieving favorable outcomes.

**Conclusion:**

Long-term follow-up is essential in patients with anterior chamber phakic IOLs due to the risk of progressive complications such as cataract formation and secondary glaucoma. Early diagnosis of glaucoma could have prevented advanced optic nerve damage in the presented patient. In complex cases with multiple potential causes of visual impairment, electrophysiological examinations may serve as a supportive tool in the differential diagnosis of optic neuropathies.

## Introduction

The prevalence of high myopia has increased substantially in recent decades and is considered a growing public health concern among young people. It has reached epidemic proportions. In addition to genetic factors, it can be caused by environmental conditions such as staying in closed rooms for a long time and extended focus on near objects, including books, computer screens, and smartphones (1–3).

The history of phakic intraocular lenses (pIOLs) dates back to 1953 (4). Although the first refractive results were encouraging, the number of corneal complications as well as increased pressure on the iris leading to chronic inflammation and retinal edema, pupil malformation and lens instability contributed to forsaking this method (5, 6).

New types of pIOLs were needed, so from the 1970s until the beginning of 2010s, various angle-supported pIOLs were introduced. The aim was to employ diverse materials and optic designs while reducing corneal complications and minimizing pressure on the iris root. Despite achieving favourable visual and refractive outcomes, the high rate of complications associated with angle-supported pIOLs led to their removal from the market (1).

In 1986, Fyodorov and Zuev began implanting IOLs with ‘‘anterior-posterior’’ placement.

The first type of this lens was referred to a ‘‘collar button’’ or ‘‘mushroom”. It was a one-piece silicone lens with a 3.2-mm optic concave on the frontal surface, projecting anteriorly through the pupil. The lens was fixated behind the iris plane by two large haptics, with a total length of 8.0 mm. The small diameter of the optic caused visual disturbances during the night-time and photophobia during daytime. The iris could not constrict beyond 4.0 mm (7).

Common complications included pupillary block glaucoma and iridocyclitis. Over time corneal decompensation, late-onset uveitis, and cataract were reported. As a result, Fyodorov decided to discontinue the study of these implants (7).

Patients with early-generation phakic intraocular lenses implanted several decades ago are now presenting with late complications that are rarely encountered in contemporary clinical practice.

The present case illustrates the diagnostic and therapeutic challenges associated with advanced glaucomatous optic neuropathy developing many years after implantation of a first-generation Fyodorov “collar-button” anterior-posterior phakic IOL. Particular attention is given to the clinical decision-making process and the choice of a minimally invasive pressure-lowering procedure in a complex surgical context.

## Case report

We present a diagnostic and therapeutic process in a challenging case of severe vision loss in myopic patient with pIOL.

A 45-year-old patient presented with worsening vision in both eyes. He had undergone first-generation Fyodorov ‘collar button’ anterior-posterior pIOL implantation 25 years earlier. He did not provide any medical documentation and claimed that he had not visited an ophthalmologist since the implantation. The patient did not report any general or ophthalmological diseases. He was not taking medications on a regular basis. He worked in an office environment without exposure to harmful factors and did not use any stimulants.

At the initial visit, the decimal corrected distance visual acuity (CDVA) on Snellen chart was 0.9 in the right eye (RE) and 1.0 in the LE. Corrected near visual acuity (CNVA) on the Jaeger chart was 0.5 in both eyes. Intraocular pressure (IOP) was 26 mmHg in the RE and 24 mmHg in the LE.

**Figure 1:**
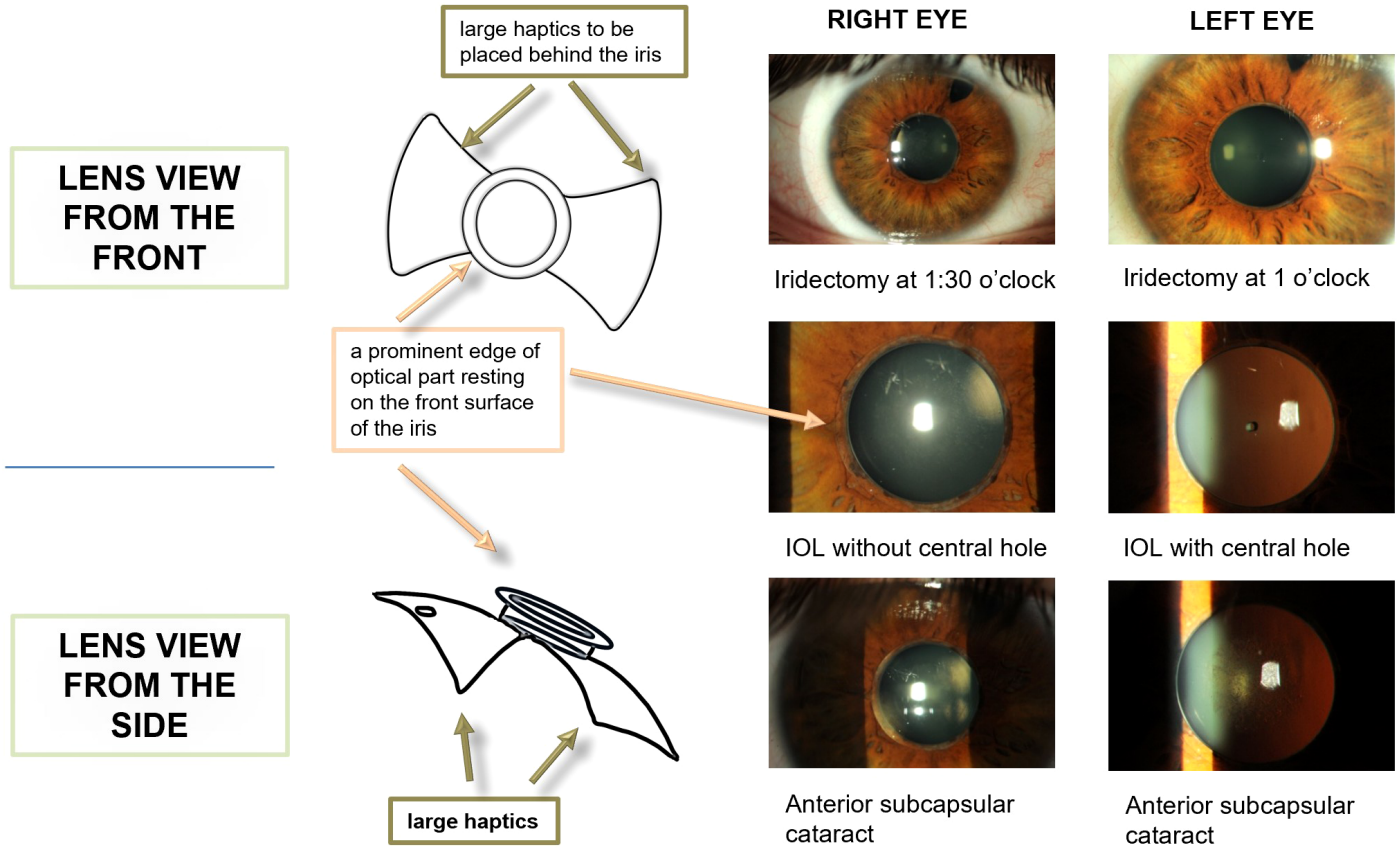
Slit lamp examination and lens design. Schematic drawing of phakic intraocular lens, front view, side view.

Slit-lamp examination revealed anterior-posterior pIOL in both eyes (**Figure 1**). The cornea was clear, and no keratic precipitates or inflammatory cells were observed in the anterior chamber. In the LE, the pIOL had an additional small aperture in the center and was more convex compared to the pIOL in the RE.

The exact reason for this difference could not be determined, as the patient did not have surgical documentation from the time of implantation; however, early models of Fyodorov phakic intraocular lenses were reported to vary slightly in their design, including the presence or absence of a central aperture.

**Figure 2:**
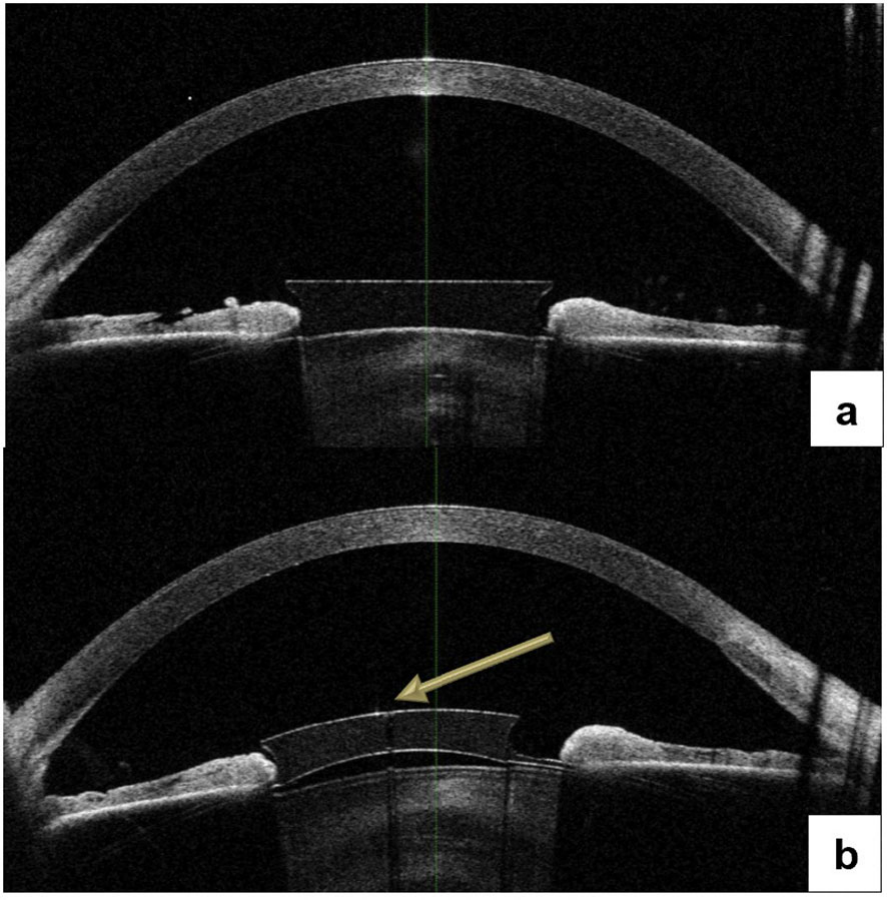
AS-OCT examination with precise visualization of two different types of Fyodorov lenses. Description: **(a)** Right eye (RE) – phakic intraocular lens without a central aperture. **(b)** Left eye (LE) – phakic intraocular lens with a central aperture (arrow). AS-OCT, anterior segment optical coherence tomography.

The anterior optical part of the pIOLs was imaged using anterior segment optical coherence tomography (AS-OCT; CASIA 2 Tomey, Nagoya, Japan), as presented in **Figure 2**.

Anterior capsular cataracts were present in both eyes. Fundus examination revealed severe glaucomatous optic neuropathy in both eyes with marked optic disc cupping (cup-to-disc ratio approximately 0.9 in both eyes), peripapillary atrophy, and no evidence of optic disc hemorrhage. Gonioscopy showed an angle width of 30 degrees with grade 3 pigmentation of the trabecular meshwork in both eyes. No photographic documentation was obtained, and the findings are therefore presented descriptively.

Static visual field (Octopus 600, Haag-Streit, Switzerland) indicated advanced scotomata in both eyes, more pronounced in the RE.

Posterior segment optical coherent tomography (PS-OCT; Zeiss, Cirrus 6000, Germany) demonstrated severe ganglion cell and retinal nerve fiber loss in both eyes (signal strength 3/10 for both eyes).

Specular microscopy performed in our center demonstrated an endothelial cell density of approximately 1500 cells/mm². Baseline endothelial cell data from the time of phakic intraocular lens implantation were not available.

**Figure 3:**
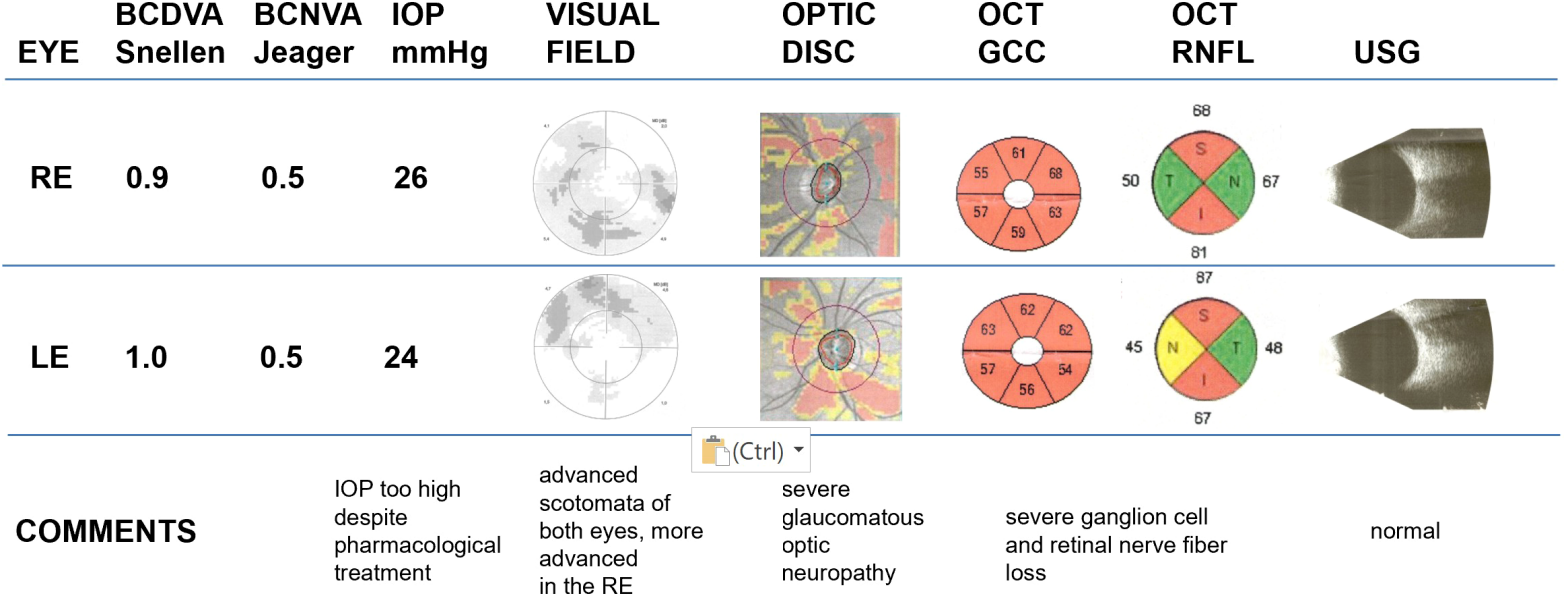
Patient’s tests summary. Description: CDVA, CNVA, IOP, visual field, optic disc appearance, PS-OCT of the GCC, optic nerve and USG. VF tests demonstrated acceptable reliability indices (fixation losses <20%, false-positive responses <15%, false-negative responses <15%). OCT signal strength: RE 3/10, LE 3/10; CDVA, best corrected distal visual acuity; CNVA, best corrected near visual acuity; IOP, intraocular pressure; PS-OCT, posterior segment optical coherence tomography; OCT GCC, optical coherence tomography, ganglion cell complex; OCT RNFL, optical coherence tomography, retinal nerve fiber layer; USG, Ultrasonography.

The summary of the undertaken tests is presented in **Figure 3**.

**Figure 4:**
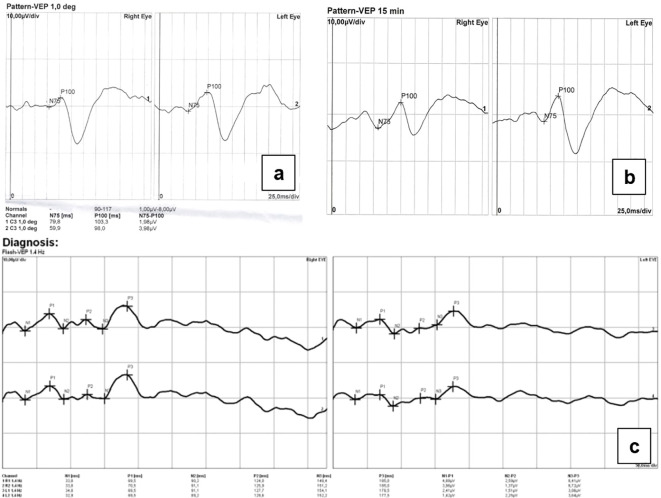
**(a)** PVEP 1.0 degree, **(b)** PVEP 15 min, **(c)** FVEP. PVEP/FVEP demonstrated reduced amplitudes of the P100/P2 wave in both eyes, more abnormal in the RE.

Pattern and flash visual evoked potentials (PVEP/FVEP) were recorded using Reti-Port electrophysiological device (Roland Consult, Germany), in accordance with the standards of the International Society of Clinical Electrophysiology of Vision (ISCEV) (8), as shown in **Figure 4**.

PVEP/FVEP demonstrated reduced amplitudes of the P100 and P2 waves, with more pronounced abnormalities in the RE. VEP findings confirmed bilateral glaucomatous optic nerve atrophy.

The patient was prescribed dorzolamide 2% and timolol 0.5% (as the fixed combination Cosopt^®^) eye drops, administered twice daily in each eye, along with brimonidine 0.2% (Alphagan^®^) eye drops, also twice daily in each eye. Prostaglandin analogues were not used due to an allergic reaction reported after their previous introduction. The patient reported good adherence to the prescribed treatment.

At the next visit, 3 weeks later, despite the use of antiglaucoma medications, IOP remained elevated, with measurements of 23 mmHg in the RE and 21 mmHg in the LE. The patient was referred to the ophthalmology department. Topical treatment was continued, and systemic treatment with acetazolamide 500 mg once daily was introduced. Due to the persistently elevated IOP in the RE (22 mmHg), a decision was made to perform a laser micropulse transscleral cyclophotocoagulation (MP-TSCPC).

During the laser procedure, the patient received only topical anesthesia without sub-Tenon’s anesthesia (e.g., lignocaine with bupivacaine), due to the risk of intraocular lens (IOL) displacement caused by iris dilation.

Following MP-TSCPC of the RE, the IOP decreased to 16 mmHg. The LE was managed pharmacologically with good results. CDVA remained at 1.0 on the Snellen chart, while CNVA was stable at 0.5 on the Jaeger chart at the 12-month follow-up.

Repeated OCT examinations during follow-up suggested thinning of the RNFL and GCC; however, due to cataract progression, signal strength was very low and the measurements were considered unreliable.

Standard automated perimetry revealed advanced visual field defects consistent with significant functional impairment; however, the presence of cataract may have affected the reliability of these findings. Therefore, electrophysiological testing played a crucial role, as it is less dependent on optical media clarity, allowing for accurate differential diagnosis and identification of the cause of visual impairment.

Longitudinal assessment of VEP demonstrated stable to improved electrophysiological responses over time (**Table 1**). Pattern VEP showed a reduction in P100 latency between September 2023 and December 2025, particularly for larger check sizes (1°), with values decreasing from 103 ms to 93 ms in the right eye and remaining relatively stable in the left eye (98 ms to 101 ms). A similar trend was observed for smaller check sizes (15′), with latency shortening in both eyes.

**Table 1:** Longitudinal parameters of VEP: PVEP (1° and 15′) and FVEP.

Examination date	Eye	PVEP				FVEP	
		1° P100 (ms)	1° N75–P100 (µV)	15′ P100 (ms)	15′ N75–P100 (µV)	P2 (ms)	N2–P2 (µV)
June 2023	R	103	1.98	117	5.04	124	1.53
	L	98	3.98	121	5.16	104	1.17
December 2025	R	93	5	110	4.22	–	–
	L	101	7.6	101	7.56	–	–
March 2026	R	–	–	–	–	83	10.35
	L	–	–	–	–	87	9.96

Concomitantly, a marked increase in N75–P100 amplitude was observed, especially for 1° stimulation (from 1.98 µV to 5.0 µV in the right eye and from 3.98 µV to 7.6 µV in the left eye), indicating enhanced cortical response. Responses to 15′ stimuli also demonstrated increased amplitudes, particularly in the left eye.

FVEP recorded in March 2026, revealed symmetric and robust responses with P2 latencies of 83 ms (right eye) and 87 ms (left eye), and high amplitudes (10.35 µV and 9.96 µV, respectively), without significant interocular asymmetry.

**Figure 5:**
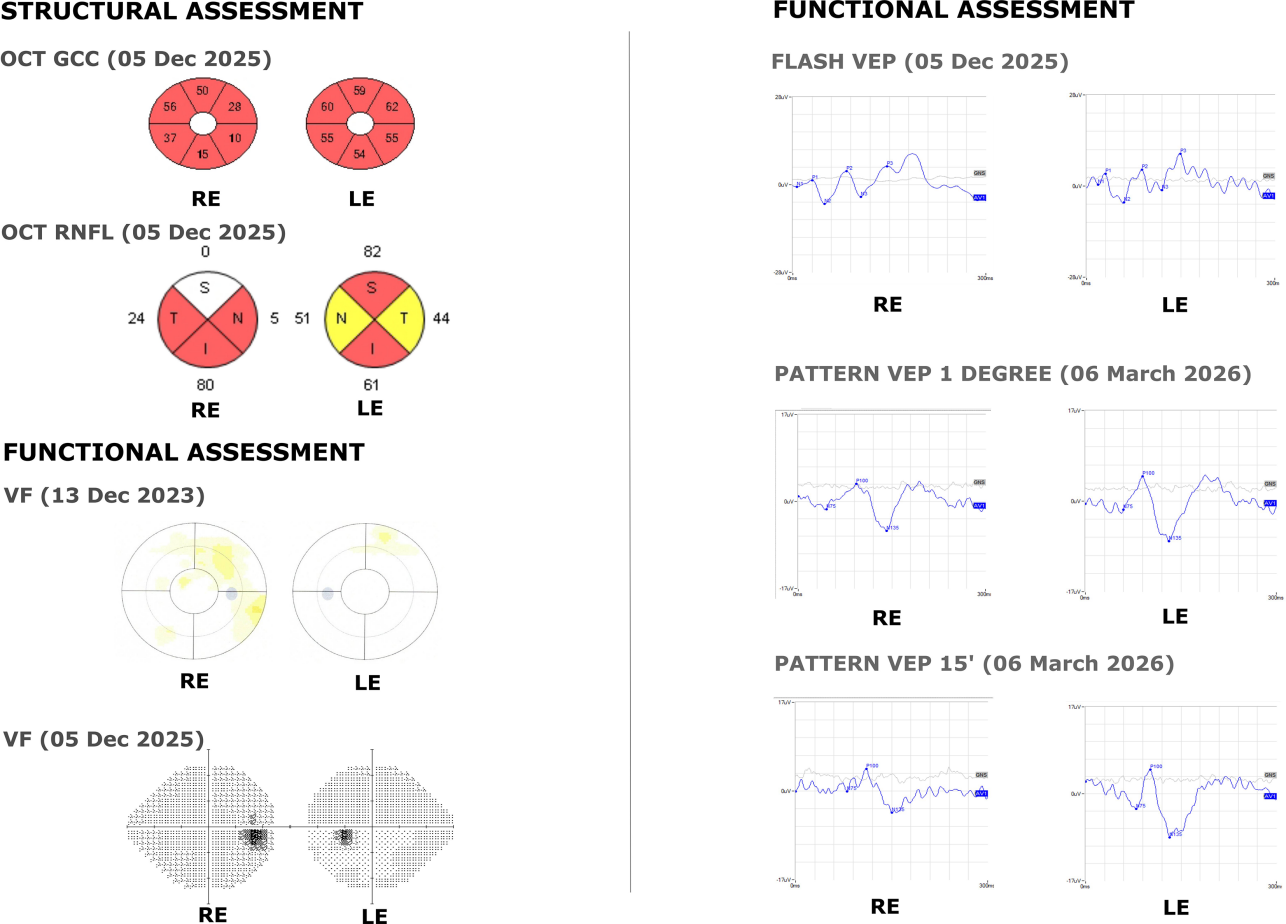
Longitudinal multimodal structural and functional assessment of advanced glaucomatous optic neuropathy. Structural assessment (left panel) includes optical coherence tomography (OCT) measurements of the ganglion cell complex (GCC) and retinal nerve fiber layer (RNFL), obtained in December 2025, demonstrating marked thinning in both eyes, consistent with advanced glaucomatous damage. Functional assessment (right panel and lower left panel) comprises standard automated perimetry (visual field, VF; December 2025), showing advanced scotomas in both eyes, as well as electrophysiological testing, including FVEP (December 2025) and PVEP (March 2026), Electrophysiological findings are consistent with significant dysfunction of the visual pathway and retinal ganglion cells. Visual field reliability indices: RE: fixation losses 0/0, false positives 0%, false negatives 2%, GHT outside normal limits, VFI 97%, MD −5.60 dB (P < 1%), PSD 1.81 dB (P < 10%). LE: fixation losses 0/0, false positives 5%, false negatives 0%, GHT outside normal limits, VFI Since the initial submission, additional follow-up examinations, including OCT imaging and comprehensive ophthalmological assessments, have been performed, confirming the stability of the clinical condition after treatment. The patient described in this report remains under regular ophthalmological follow-up, and ongoing clinical observations will provide further insight into the long-term outcomes in this case. 95%, MD −2.91 dB (P < 1%), PSD 3.66 dB (P < 0.5%). The reliability indices were within acceptable limits in both eyes. OCT signal strength: RE 1/10, LE 1/10; RE, right eye; LE, left eye; OCT, optical coherence tomography; GCC, ganglion cell complex; RNFL, retinal nerve fiber layer; VF, visual field; VEP, visual evoked potentials.

Electrophysiological testing, including PVEP and FVEP confirmed severe dysfunction of the visual pathway and retinal ganglion cells. Longitudinal visual field and OCT examinations obtained during follow-up are presented in **Figure 5** and demonstrate structural and functional stability during the observation period.

The clinical timeline of diagnostic and therapeutic events is summarized in **Table 2**.

**Table 2:** Clinical timeline of diagnostic and therapeutic events.

Time	Clinical event
*June 2023 – Initial visit*	*IOP was 26 mmHg in the RE and 24 mmHg in the LE. VF and OCT examinations were performed. Antiglaucoma treatment was initiated (topical dorzolamide/timolol fixed combination and brimonidine).*
3 weeks later	Systemic acetazolamide was added.
July 2023	IOP remained elevated; MP-TSCPC was performed in the RE.
Follow-up visits every 3–6 months until March 2026 (last visit).	CVA decreased to 0.8 in the RE and remained stable in the LE. IOP was stable and controlled in both eyes.

## Discussion

Despite the many advantages of Fyodorov pIOL and its popularity in 1990’s (9) refractory glaucoma developed in this case.

In the present case, the clinical findings were most consistent with secondary open-angle glaucoma associated with phakic intraocular lens implantation and pigment dispersion.

Although secondary glaucoma after phakic IOL implantation has been previously reported, cases involving historical Fyodorov “collar-button” lenses with such long follow-up are rarely described in contemporary literature.

In general, complications are infrequent but heavily dependent on the location and positioning of the pIOL. Introducing foreign materials into the eye can cause intraocular tissue injuries or obstruct the outflow of aqueous humor from the chamber, potentially leading to elevated IOP (10, 11). Additionally, individuals with myopia are at a higher risk of developing cataract at an earlier age. PIOLs might also accelerate the development of cataract, leading to reduced accommodation and diminished visual acuity, which may necessitate the removal of the pIOL in conjunction with cataract surgery (12). Cataract formation following uncomplicated pIOL implantation thought to be associated with inflammation, a complication observed across all pIOL types. However, patients with posterior chamber pIOLs are particularly prone to cataract formation. The contact and friction between the pIOL and the crystalline lens in these cases pose a heightened risk of developing anterior subcapsular cataract, often requiring cataract surgery at a younger age. The presence of adhesions between the crystalline lens and its surrounding capsule further increases the complexity of surgical procedures (13, 14). The reduction in endothelial cell count is the second most frequent complication associated with pIOL implantation, most commonly associated with anterior chamber pIOLs. Additionally, iris-fixated and posterior chamber pIOLs may cause issues such as pigment dispersion and pupil block (15).

The patient presented with worsening vision in both eyes. It was challenging to determine the exact cause of the vision deterioration. Initially, anterior subcapsular cataract, optic neuropathy, or a combination of both were considered as potential causes.

Because topical and systemic therapy failed to adequately control intraocular pressure, selection of an appropriate surgical procedure became necessary.

In patients with historical phakic intraocular lenses presenting with glaucoma and cataract, several management strategies may be considered. These include explantation of the phakic IOL combined with cataract surgery, combined cataract–glaucoma procedures, or less invasive pressure-lowering interventions such as micropulse transscleral cyclophotocoagulation.

In the present case, the decision to perform MP-TSCPC was influenced by several factors, including the preserved central visual acuity, the technical complexity of removing first-generation Fyodorov lenses, and the patient’s reluctance to undergo extensive intraocular surgery.

Removal of the pIOLs was considered. However, the patient declined this option, citing the large size of the lenses and the significant surgical intervention required. Meanwhile, selecting an alternative surgical method to effectively lower intraocular pressure remained problematic.

Penetrating glaucoma procedures could be complicated by hypotony and the need for cycloplegics, which were contraindicated because the risk of pIOLs displacement with pupil dilatation. Additionally, hypotony and anterior chamber flattening could lead to corneal decompensation by causing the optical part of the artificial lens to protrude through the iris.

In such cases, several treatment strategies may be considered. One option is explantation of the phakic intraocular lens combined with cataract surgery, which may remove a potential source of pigment dispersion and improve anterior chamber anatomy, although the procedure may be technically challenging in eyes with early-generation pIOLs. Another approach involves combined cataract and glaucoma surgery, particularly in patients with uncontrolled intraocular pressure or advanced glaucomatous damage (11). Alternatively, less invasive pressure-lowering procedures, such as micropulse transscleral cyclophotocoagulation, may be used as an initial or temporizing intervention to reduce intraocular pressure while avoiding extensive intraocular surgery. The choice of treatment should therefore be individualized, taking into account the patient’s clinical condition, surgical risks, and preferences.

Given its minimally invasive nature, the MP-TSCPC procedure was selected.

If clinically indicated in the future, explantation of the phakic intraocular lens combined with cataract surgery may still be considered as a subsequent treatment option.

Following the MP-TSCPC procedure, a clinically meaningful reduction in intraocular pressure was observed. However, given the nature of a single case report, these findings should be interpreted with caution and cannot be considered definitive evidence of treatment efficacy. Diurnal IOP measurements were not available.

A longitudinal multimodal structural and functional assessment was performed to evaluate the extent of glaucomatous damage.

The endothelial measurements remained stable during subsequent follow-up visits.

Additional imaging modalities, such as ultrasound biomicroscopy or flare measurements, could further support the mechanistic interpretation.

Earlier visual field examinations were performed using the Octopus perimeter.

The most recent follow-up tests were obtained with a Humphrey Visual Field analyzer, and the reliability indices from these examinations are reported accordingly in the legend of **Figure 5**.

The patient requires regular ophthalmological follow-ups to monitor IOP and assess the progression of glaucoma and cataract. Calculating the power of the future intraocular lens, to be implanted during the removal of the pIOL and the patient’s cataractous lens, may present a significant challenge. Furthermore, the surgical risks associated with this procedure, as well as the potential for postoperative complications, must be carefully evaluated.

The findings from both pVEP and fVEP suggest improvement of visual pathway conduction, as evidenced by reduced or stable P100 latencies and significantly increased amplitudes over time.

In complex clinical situations, electrophysiological testing may provide valuable complementary information in the differential diagnosis of optic neuropathies, particularly when standard structural and functional assessments are limited.

Further long-term follow-up will be important to determine whether sustained intraocular pressure control achieved after MP-TSCPC will translate into long-term structural and functional stability.

## Conclusions

Patients with historical phakic intraocular lenses require long-term ophthalmological follow-up due to the risk of late complications, including cataract formation and secondary glaucoma. The present case highlights the diagnostic and therapeutic challenges associated with advanced glaucomatous optic neuropathy developing many years after implantation of a first-generation Fyodorov “collar-button” phakic intraocular lens.

In selected cases, minimally invasive procedures such as MP-TSPC may represent a useful option for intraocular pressure control when extensive intraocular surgery is not feasible or not accepted by the patient.

Electrophysiological testing may serve as a supportive tool in the differential diagnosis of optic neuropathies in complex clinical situations.

## Data Availability

The original contributions presented in the study are included in the article. Further inquiries can be directed to the corresponding authors.
